# Tocilizumab in Large Vessel Vasculitis – Different Routes of Administration

**DOI:** 10.2174/1874312901812010152

**Published:** 2018-08-31

**Authors:** Marc Schmalzing, Ottar Gadeholt, Michael Gernert, Hans-Peter Tony, Eva C Schwaneck

**Affiliations:** Focus on Rheumatology / Clinical Immunology, Department of Internal Medicine II, University of Würzburg, 97080 Wurzburg, Germany

**Keywords:** Tocilizumab, Giant cell arteritis, Takayasu arteritis, Route of administration, Infections, Subcutaneous, Intravenous

## Abstract

**Background:**

Tocilizumab is increasingly used in the treatment of large vessel vasculitis with recent approval for giant cell arteritis.

**Objective:**

To determine the efficacy and safety of tocilizumab in large vessel vasculitis in a real-life setting using different routes of administration.

**Methods:**

Retrospective analysis of consecutive patients at a tertiary rheumatology department who received tocilizumab for large vessel vasculitis.

**Results:**

A total of 11 patients were treated with tocilizumab (8 giant cell arteritis, 2 large vessel vasculitis associated with rheumatoid arthritis, 1 Takayasu arteritis) after a median of 2 other steroid-sparing agents (range 1-4). Of these, 9 received tocilizumab as salvage therapy for active vasculitis and 2 due to the toxicity of their former steroid-sparing medication. After a mean follow-up of 23 months 7 patients were in remission as to vasculitis under a mean prednisolone dose of 1.7 ± 1.5 mg; one patient relapsed after long term remission having discontinued tocilizumab for elective surgery; one patient stopped tocilizumab after attributable infectious complications, and two patients died: one due to complications of vascular surgery, probably not attributable to tocilizumab; and the other due to sepsis secondary to sigmoiditis. Only 3 relapses occurred under continuous tocilizumab treatment. In all these 3 cases, renewed remission could be achieved by switching from subcutaneous (162 mg qw) to intravenous tocilizumab (8mg/kg q4w).

**Conclusion:**

Tocilizumab is efficacious in patients with large vessel vasculitis in a real-life situation. Safety appears to be acceptable, but infectious complications have to be considered. Intravenous tocilizumab may be used in patients who relapse under subcutaneous application.

## 1 INTRODUCTION

Takayasu Arteritis (TAK) and Giant Cell Arteritis (GCA) are the two major variants of Large Vessel Vasculitis (LVV). LVV can also occur in association with other inflammatory rheumatic diseases [1].TAK mainly involves the aorta and large arterial branches leading to ischemic complications of limbs and brain, whereas symptoms in GCA are usually caused by the predominant involvement of temporal arteries or other medium-sized extracranial arteries. Both diseases can cause severe morbidity and mortality.

Glucocorticoids still constitute the mainstay for treatment of LVV, but can cause severe side effects, and are associated with a high relapse rate, when used as monotherapy [2].Therefore, in 2009 the European League against Rheumatism (EULAR) recommended considering upfront immunosuppressive steroid-sparing medication in LVV in addition to glucocorticoid therapy [3]. Common drugs to consider in this context would be methotrexate, azathioprine, leflunomide, cyclophosphamide, and more recently biological Disease Modifying Anti-Rheumatic Drugs (DMARDs) like TNF inhibitors, abatacept, ustekinumab, and tocilizumab.

Tocilizumab is a humanized immunoglobulin G1 kappa monoclonal antibody that blocks signalling by binding to the alpha chain of the human interleukin-6 receptor, and was approved for the treatment of rheumatoid arthritis, systemic or polyarticular juvenile idiopathic arthritis, and recently, giant cell arteritis [4].

There are several studies which show the efficacy of tocilizumab in GCA regarding remission rate, relapse free survival, and steroid-sparing [5-15]. In these studies, tocilizumab was exclusively used as an intravenous application, with the exception of the recently published, multicentric, randomized GiACTA trial, which led to approval of subcutaneous tocilizumab in giant cell arteritis. In TAK - being the rarer disease - literature on the efficacy of tocilizumab is less conclusive.

Although there is ample evidence of efficacy of tocilizumab in GCA, many questions as to the efficacy in other forms of LVV, route of administration, or safety remain.

The aim of this retrospective analysis was to evaluate efficacy and safety of tocilizumab in different forms of large vessel vasculitis in a real-life setting. Since some patients received tocilizumab both intravenously (TCZ-IV) and subcutaneously (TCZ-SC), an attempt to compare the efficacy of these different routes of application could be undertaken.

## 2 MATERIALS AND METHODS

A retrospective review of medical records of consecutive patients with large vessel vasculitis at our center, who received tocilizumab, was conducted. The following data were retrieved from an electronic database: patient characteristics, description of vasculitis (C-Reactive Protein (CRP), Erythrocyte Sedimentation Rate (ESR), disease duration, imaging modality documenting vasculitis, complications) and treatment (pretreatment, glucocorticoid dose, adverse events). Descriptive statistics were reported as mean and Standard Deviation (SD). Further statistical analyses were not undertaken due to low number of patients.

For patients with active disease, the mean glucocorticoid dose prior to tocilizumab and at visits at month 3 and month 6 were assessed. Follow up lasted as long as patients received tocilizumab treatment. Glucocorticoid dose at last follow up was assessed for all patients. Physician-assessed vasculitis relapses were recorded (defined as the presence of unambiguous symptoms of active vasculitis or progression in imaging). Acute phase reactants CRP and ESR were not evaluated as outcome parameters, since tocilizumab led to false negative results as expected. Imaging modalities which were used to monitor disease activity were Color Doppler Ultrasound (CDU), fluorodeoxyglucose - Positron Emission Tomography/Computerised Tomography scan (PETCT) and Magnetic Resonance Imaging (MRI). Efficacy of treatment was defined as achievement of sustained remission, *i.e*. normalization of vessel imaging, and absence of symptoms.

Due to the observational, retrospective data analysis, approval from the local ethics committee was not required by German law.

## 3 RESULTS

### 3.1 Baseline Characteristics

In total, 11 patients received tocilizumab (TCZ-IV 8mg/kg q4w or TCZ-SC 162 mg qw) for the diagnosis of large vessel vasculitis from July 2012 to July 2017 (patient characteristics: Table **1**): 8 patients with giant cell arteritis, 2 patients with large vessel vasculitis associated with rheumatoid arthritis, and 1 patient with Takayasu arteritis.

In 9 patients tocilizumab was started due to active vasculitis after failure of a median of 2 immunosuppressive agents (range 1-4), including Methotrexate (n=9), leflunomide (n=7), cyclophosphamide (n=5), or azathioprine (n=4). Active vasculitis directly before the start of tocilizumab was properly documented in all patients by CRP (n=6), ESR (n=7), CDU (n=5), MRI (n=4), and PETCT (n=3). The other 2 patients without active vasculitis were started on TCZ-SC due to side effects of their former steroid-sparing medication.

In 7 patients, tocilizumab was started as a subcutaneous application. Of the 4 other patients who started tocilizumab intravenously, one stayed on TCZ-IV (number 6), while three switched from TCZ-IV to TCZ-SC in remission for logistic reasons (two after 8 weeks, and one after 27 months).

### 3.2 Efficacy

Patients with active vasculitis had a mean CRP of 5.02 +- 5.87 mg/dl and a mean ESR of 58.2 +-45.9 mm/h. As expected, CRP and ESR normalized after the initiation of tocilizumab, and stayed so throughout the whole treatment period in all patients except for one female patient with rheumatoid arthritis, who had an arthritic relapse after 6 months of TCZ-SC (CRP 1.50 mg/dl). The MRI at that time did not show active vasculitis of the aorta.

In addition to serological markers, the disease course of patients with active vasculitis was monitored by clinical parameters (n=9), CDU (n=6), MRI (n=3), and PETCT (n=1).

In the 8 patients with active vasculitis who could be evaluated, mean daily prednisolone dose at the time of flare before steroid pulse and the start of tocilizumab was 11.3 ± 7.7 mg. Mean prednisolone dose of the steroid pulse was 185.8 ± 336.1 mg (range 5 - 1000 mg). Mean prednisolone dose was 10.2 ± 5.9 mg after 3 months of tocilizumab, and 9.2 ± 8.6 mg after 6 months, respectively. One patient (number 5) with active vasculitis refused to reduce his prednisolone dose below 75 mg daily for fear of blindness and was excluded from the analysis.

After a mean follow up of 23 months (range 4-60) 8 of 11 patients were still under tocilizumab treatment (4 TCZ-IV, 4 TCZ-SC), with a mean prednisolone dose of 4,0 mg (± 6.6 mg). Excluding the patient (number 9) who relapsed probably because of perioperative termination of TCZ, last mean prednisolone dose was 1.7 ± 1.5 mg in the 7 patients in remission as to vasculitis. Reasons for discontinuation of TCZ in the other 3 patients are described below (safety chapter).

Of the 9 patients with active vasculitis before initiating TCZ 3 relapsed with vasculitis (all under TCZ-SC, number 9-11). In all 3 patients, TCZ-SC was switched to TCZ-IV, which led to a marked response in all patients. Since this response may speak in favor of a superior efficacy of TCZ-IV, we present these three cases in more detail.

#### 3.2.1 Patient 9

In a 71-year-old, female patient with GCA, MRI showed vasculitis of the abdominal aorta and both common iliac arteries. Response to methotrexate alone or combined with leflunomide, and cyclophosphamide was inadequate, upon which TCZ-IV was commenced. After two initial intravenous applications, TCZ was switched to TCZ-SC. Prednisolone was tapered to 5 mg within 4 months without symptoms indicating relapse. The patient relapsed after seven months, with cervical tenderness, signs of aortitis on MRI and vessel wall thickening of the carotid arteries (daily prednisolone dose at relapse: 5 mg). TCZ-SC was switched back to TCZ-IV, and prednisolone dose was increased to 30 mg with consecutive tapering. 3 months later, under 10 mg of prednisolone, wall thickening of the carotid arteries and of the aorta markedly decreased in CDU, and MRI respectively. The patient was free of clinical symptoms and remained in remission for 9 months (prednisolone dose 5 mg), until tocilizumab was stopped in preparation of valve surgery for progressive aortic regurgitation. 4 weeks after surgery, tocilizumab was restarted and prednisolone dose increased to 20 mg due to relapse (aortic histology, CRP).

#### 3.2.2 Patient 10

In a 47-year-old, female patient with GCA and anterior ischemic optical neuropathy, MRI and CDU showed halo and increased wall thickness of both ACC. Steroid-sparing treatment with methotrexate, leflunomide, and azathioprine had to be stopped due to toxicity. Intravenous cyclophosphamide (cumulative dose: 8000 mg) was ineffective. Therefore, TCZ-SC was started. Within 6 months prednisolone could be reduced to 5 mg, and CDU aspect became normal. After 10 months of TCZ-SC treatment, the patient relapsed with severe cervical tenderness responsive to prednisolone (daily prednisolone dose before pulse 5 mg). Therefore, TCZ-SC was switched to TCZ-IV. After a further follow up of 24 months, the patient is still in remission after tapering prednisolone to 3 mg with normal CDU.

#### 3.2.3 Patient 11

In a 21-year-old, female patient with TAK, CDU showed vessel wall thickening of both ACC, occluding the right one, of both internal and external carotid arteries, the left subclavian artery with occlusion, and the left vertebral artery with occlusion. She relapsed after 2 months of treatment with methotrexate and prednisolone, wherefore TCZ-SC was added and prednisolone increased from 15 to 100 mg. While tapering prednisolone in the following 4 months, the patient suffered two minor and one major relapse (severe cervical tenderness and progressive wall thickening in CDU), treated by prednisolone pulses. Prednisolone was increased from 15 mg to 100 mg and TCZ-SC was switched to TCZ-IV. Since the switch, no further relapse occurred during follow up of 20 months, and prednisolone could be tapered to 4 mg.

### 3.3 Safety

Three patients suffered infectious complications under TCZ treatment:

One patient (number 3) had to stop tocilizumab after 4 months due to Campylobacter-induced colitis and oropharyngeal candidiasis under a concomitant prednisolone dose of 10 mg.

The second patient (number 4) developed cellulitis of her right foot after one application of TCZ-SC under 5 mg prednisolone daily. After antibiotic therapy tocilizumab could be restarted, which she received for one more year. TCZ-SC was then stopped in preparation for vascular surgery of progressive aortic aneurysm. TCZ could not be restarted due to severe complications after surgery, of which she died 7 months later.

The third patient (number 5) died from sepsis shortly after surgery for a pertrochanteric fracture after 4 months of TCZ treatment. *Escherichia coli* was found in several blood cultures, and wall thickening of the sigma was detected in CT scan. This was the aforementioned patient who did not lower his prednisolone dose below 75 mg due to fear of blindness.

No other side effects could be attributed to tocilizumab in all patients.

## 4 DISCUSSION

In this retrospective analysis, we confirm the efficacy and safety of tocilizumab in different forms of large vessel vasculitis, either given intravenously or subcutaneously, even in a heavily pretreated patient population. Seven out of eleven patients still showed good disease control with low prednisolone dose under continuous tocilizumab, at last, follow up (mean follow up of about 23 months, range 4-60 months).

Although there was good disease control in our study with tocilizumab treatment, the mean steroid-sparing effect in the first 6 months was only marginal. In the majority of patients, prednisolone could be reduced to an acceptable level according to EULAR guidelines only after longer follow-up [16]. The fact that 56% of patients in the tocilizumab arms in the GiACTA trial had sustained remission in week 52 with a prednisone tapering regimen of merely 26 weeks, would suggest a very strong steroid-sparing effect with a high rate of prednisone discontinuation. This was not necessarily the case in our heavily pretreated population arguing in favor of an important role of a glucocorticoid maintenance therapy in combination with tocilizumab.

So far, there is no evidence that intravenous application of tocilizumab shows superior efficacy compared to subcutaneous application in large vessel vasculitis. However, in our cohort three patients, who relapsed under TCZ-SC, showed good disease control after switching to TCZ-IV, although prednisolone was then tapered to an even lower dose than under TCZ-SC. The disease course of these three patients may speak in favor of a better efficacy of TCZ-IV compared to TCZ-SC.

This postulated superiority of TCZ-IV could be explained by different pharmacokinetics. Studies in RA show comparable serum trough levels for both TCZ-IV and TCZ-SC [17], but a higher area under the curve (AUC) for TCZ-IV [18]. Furthermore, it was shown that the efficacy of TCZ-SC could be worse in patients with high body weight [19]. Only one of the three patients, who relapsed in our study under TCZ-SC, was obese with a body weight of 100 kg, the two other patients weighed only 61 and 57.5 kg respectively. Thus, obesity cannot explain the inferior efficacy of TCZ-SC in our patients.

Another explanation for the different efficacy of TCZ-IV and TCZ-SC could be disease-related. Response to interleukin-6-receptor-inhibition by tocilizumab with respect to elevation of interleukin-6 or soluble-interleukin-6-receptor in patients sera can depend on the disease entity. This was shown in a study of rheumatoid arthritis and Castleman’s disease, respectively [20]. So far, there are no similar observations concerning large vessel vasculitis.

The inferior disease control under TCZ-SC, which the three patients applied themselves, may in principle be due to worse adherence. Nevertheless, the patients in question did not show any signs of lower adherence, they requested their tocilizumab prescriptions in time, ESR and CRP were always suppressed as expected, they had no difficulty with subcutaneous application, and they all showed a credible positive attitude towards tocilizumab due to fear of ischemic complications. Even in the unlikely case of better disease control under intravenous tocilizumab being caused by adherence issues, our experience would argue in favor of using the more reliable intravenous application in patients with active vasculitis prone to ischemic complications.

Whether TCZ-IV is more effective in large vessel vasculitis than TCZ-SC, should be a matter of future research.

With regard to safety, there were three severe infections in our eleven patients. One patient had to stop tocilizumab due to infectious colitis, whereas another patient could restart tocilizumab after antibiotic treatment. One patient died from sepsis, but took a high concomitant prednisolone dose. Of note, the most likely source for sepsis of the deceased patient was sigmoiditis. At least in RA patients a higher risk of lower intestinal tract perforations of tocilizumab compared to other biological DMARDs was found in studies [21]. This finding was not confirmed in the GiACTA trial. Nevertheless, this signal would call for large prospective studies in a non-interventional real life setting to evaluate safety in a population which is highly problematic as to infectious risk. Our patient population was mainly elderly, had a high cumulative dose of prednisolone, and suffered from multiple comorbidities (diabetes, osteoporosis, heart failure, renal failure, pulmonary arterial hypertension, diverticulosis), which would account for the relatively high rate of severe infections.

Our study has several weaknesses. The patient number is low and patients suffered from different forms of large vessel vasculitis, which renders the study population heterogeneous. Nevertheless, the excellent response in almost all patients, and the disease control of three patients after switching from TCZ-SC to TCZ-IV could be a basis for further studies comparing efficacy of TCZ-SC *versus* TCZ-IV in GCA and TAK, respectively.

## CONCLUSION

In summary, tocilizumab constitutes an efficacious treatment option even for heavily pretreated patients with large vessel vasculitis in a real-life situation. Safety seems to be acceptable, but infectious complications have to be considered. This report suggests that switching from subcutaneous to intravenous tocilizumab may achieve better disease control.

## Figures and Tables

**Table 1:** Patient characteristics.

Patient number	Gender	Age/yrs	Weight at start of TCZ	Disease	Follow up (FU)/mo	Indication for TCZ	Pred. before TCZ/mg*	Pred. at last FU	Specifics
1	m	62	77	GCA+PMR	25	toxicity of pretreatment	10	1,5	-
2	f	77	75	GCA+PMR	19	toxicity of pretreatment	5	1	-
3	f	61	62	GCA	4	active vasculitis	7,5	10	Colitis, switch to abatacept
4	f	62	64	LVV+RA	20	active vasculitis	5	7,5	Died in remission after aortic arc replacement
5	m	80	92	GCA+PMR	4	active vasculitis	100	100	Died of E.coli sepsis with sigmoiditis
6	f	66	69	LVV+RA	45	active vasculitis	5	2,5	-
7	f	52	60	GCA+PMR	60	active vasculitis	25	0	-
8	m	78	81	GCA+PMR	17	active vasculitis	17,5	0	-
		Switch from TCZ-SC to TCZ-IV due to relapse:							Prednisolone reduction (dose before pulse / dose at last follow-up under TCZ without relapse)
9	f	71	61	GCA	17	active vasculitis	5	20	5 mg / 5mg after 9 mo, relapse after perioperative stop of TCZ
10	f	47	100	GCA	24	active vasculitis	7,5	3	5 mg / 3 mg after 24 mo
11	f	21	57,5	TAK	20	active vasculitis	17,5	4	15 mg / 4 mg after 20 mo

*In patients with active vasculitis only dose before treatment intensification is given, not a dose of prednisolone pulse. RA=Rheumatoid Arthritis, GCA=Giant Cell Arteritis, PMR=Polymyalgia Rheumatic, LVV=Large Vessel Vasculitis, TAK=Takayasu Arteritis

## LIST OF ABBREVIATIONS

ACC: = Common carotid artery/arteries
CDU: = Color Doppler Ultrasound
CRP: = C-Reactive Protein
CT: = Computerised Tomography scan
ESR: = Erythrocyte Sedimentation Rate
EULAR: = European League Against Rheumatism
DMARDs: = Disease Modifying Anti-Rheumatic Drugs
GCA: = Giant Cell Arteritis
LVV: = Large Vessel Vasculitis
MRI: = Magnetic Resonance Imaging
PETCT: = Positron Emission Tomography/Computerised Tomography scan
PMR: = Polymyalgia Rheumatica
RA: = Rheumatoid Arthritis
SD: = Standard Deviation
TAK: = Takayasu Arteritis
TCZ: = Tocilizumab
TCZ-IV: = Intravenous Tocilizumab
TCZ-SC: = Subcutaneous Tocilizumab
